# The effectiveness of 20 μg hepatitis B vaccine used for the prevention of HBV vertical transmission

**DOI:** 10.1038/s41598-022-15744-z

**Published:** 2022-07-11

**Authors:** Guo Yonghao, Chen Yanping, Dou Qiaohua, Feng Daxing, Zhang Yanyang, Zhao Dongyang, Guo Wanshen

**Affiliations:** 1 Henan Provincial Center for Disease Control and Prevention, Zhengzhou, 450016 China; 2 College of Public Health, Zhengzhou University, Zhengzhou, 450001 China

**Keywords:** Viral hepatitis, Hepatitis B, Epidemiology

## Abstract

To evaluate the efficiency of a 20 μg hepatitis B vaccine(HepB) for disease prevention in two counties in Henan Province, China. A questionnaire was designed to examine the information of hepatitis B surface antigen (HBsAg) positive pregnant women, and their blood samples were collected to test for hepatitis B e antigen (HBeAg), hepatitis B e antibody, and hepatitis B virus (HBV) DNA. Three doses of 20 μg HepB and one dose of hepatitis B immune globulin(HBIG) were administered to newborns. Blood samples were collected from children one month after their complete immunization to test for HBsAg and hepatitis B surface antibody(HBsAb). A total of 419 HBsAg positive-pregnant women and 430 newborns were investigated. The average age of pregnant women was 29.6 ± 4.3 years, with an HBeAg positive rate of 29.1% (122/419). All newborns received their first dose of 20 μg hepatitis B vaccine and 100 IU HBIG within 12 h after birth. Six infants (1.9%, 6/319) tested positive for HBsAg and negative for HBsAb after one month of receiving the three basic doses of HepB. The geometric mean concentration(GMC) of HBsAb-positive infants was 861.6 mIU/mL, and their HBsAb antibody titers decreased with age. Immunization of children born to HBsAg-positive mothers with 20 μg HepB got the satisfactory effect on preventing mother-to-child transmission.

Hepatitis B virus (HBV) infection remains a serious global public health problem^1^. Persistent HBV infection can cause chronic liver damage, which can lead to cirrhosis and hepatocellular carcinoma. In 2015, the World Health Organization (WHO) estimated that around 257 million people worldwide had chronic HBV infection (defined as hepatitis B surface antigen-positive) and that around 887, 000 people died of liver fibrosis or liver cancer caused by hepatitis B infection^2^.

Mother-to-child transmission (MTCT) is the main way of HBV transmission. About 90% of infants infected with HBV will develop chronic hepatitis B if no vaccination is done, which is substantially higher than horizontal transmission^3,4^. The chronic infection rate is 30–50% for children under the age of six, and 5% for adults. MTCT accounts for around 50% of new hepatitis B infections in countries with high prevalence, and one-third in countries with low prevalence^5,6^. Therefore, prevention of mother-to-child transmission (PMTCT) is essential to stop vertical transmission and reduce the burden of the chronic hepatitis B (CHB). In order to prevent MTCT of hepatitis B, WHO recommends that the hepatitis B vaccine (HepB) should be given within 24 h after birth, even in areas with a low prevalence of hepatitis B. Infants born to hepatitis B surface antigen (HBsAg) positive mothers, especially those who are hepatitis B e antigen (HBeAg) positive, may benefit more from receiving HepB and hepatitis B immunoglobulin (HBIG) shortly after birth^7^.

The first plasma-derived HBV vaccines were manufactured under the name Heptavax B (Merck) and Hevac B (Institut Pasteur) in 1982. Four years later, the first genetically engineered hepatitis B vaccine was developed with recombinant HBsAg. The yeast-derived recombinant vaccines are manufactured by expression of HBsAg protein in genetically engineered yeast cells that contain the S gene^8^. Because lacking glycosylation in yeast, mammalian cell-derived vaccines are produced which contained glycosylated pre-S1 and pre-S2 proteins, in addition to the major HBsAg protein^9^. Currently, hepatitis B vaccines are formulated to contain 5–60 μg of recombinant HBsAg protein and 10–20 μg vaccines was often used for newborns with HBsAg-positive mother.

In China, a national survey in 2006 reported that the prevalence of HBsAg among women of childbearing age was 7.6%^10^, while a recent study on 15 million couples in rural China revealed a 5.2% HBsAg seropositive rate among women aged 15–49^11^. In certain areas of southeast China, the HBsAg-positive rate among women of childbearing age even exceeded 10%^12^. In 2012, the Chinese government began to implement the HIV-syphilis-HBV prevention program. All newborns born to HBsAg-positive mothers were eligible to receive three doses of HepB and one dose of HBIG (100 IU).

There are two types of HepB available for newborns in China, each with a different HBsAg content: 10 μg and 20 μg. The 10 μg vaccine is produced using yeast or China hamster oocytes (CHO) as the expression vector, while the 20 μg vaccine is produced using CHO as the expression vector. In this study, a prospective cohort study was conducted to examine the efficacy of 20 μg HepB for PMTCT.

## Results

### Characteristics of the pregnant women and their infants

**Figure 1:**
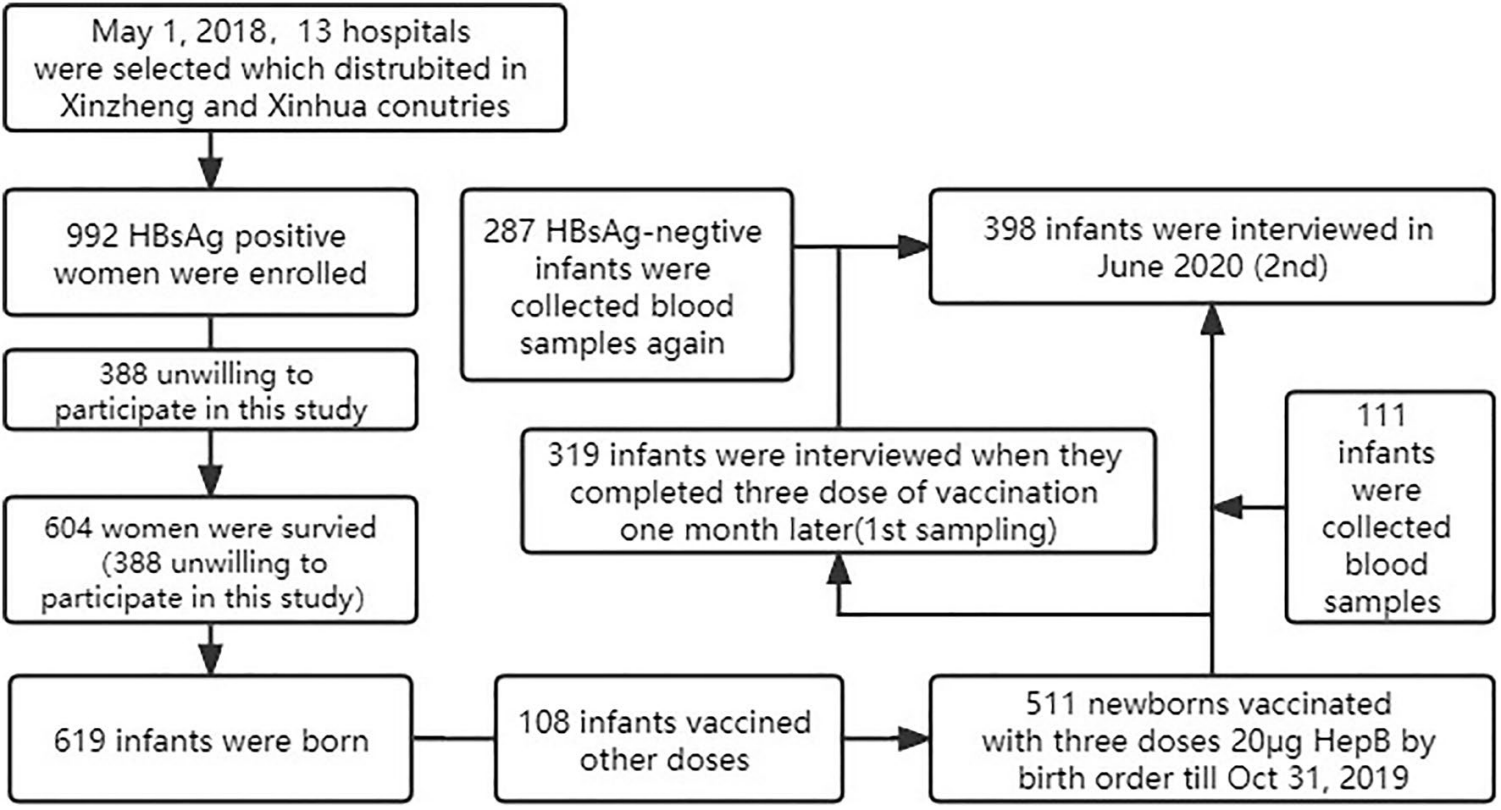
The sample flow chart of this study.

A total of 992 HBsAg positive pregnant women who delivered in 13 hospitals located in the two counties between May 1, 2018, and October 31, 2019. Among them, 604 (60.1%, 604/992) were interviewed when upon arrival at the hospital for delivery, and a total of 619 infants were born, with 511 receiving three doses of 20 μg HepB between one and six months apart. The blood samples of 319 infants were collected one month later, after they had completed three doses of vaccine, to test for HBsAg and HBsAb. In order to estimate the HBsAb variation of infants, blood samples of 398 infants, who had been vaccinated with 20 μg CHO HepB, were collected in June 2020, which included 287 infants who had their sero-samples collected for the first time. In total, 419 HBsAg-positive women and their 430 infants were analyzed (Fig. 1). The average age of the women was 29.6 ± 4.3 years, with the oldest and youngest women being 47 and 19 years old, respectively. Those with college education or above accounted for 54.9% (230/419). The oldest child was 24 months old, while the youngest child was seven months old. Ethnic Han women accounted for 99.1% (415/419) for the total, whereas female farmers for 24.1% (101/419). Among the women, 21.5% (90/419)were aware that they were infected with HBV in the previous year. Furthermore, 27.4% (115/419) of them had received antiviral therapy during pregnancy (Table 1). Twenty-five of the pregnant women gave birth before 37 weeks, and 14 out of the 270 pregnant women who filled out ALT values had ALT values higher than 40.

**Table 1:** General information of the HBsAg positive pregnant women.

Catgory	HBV DNA (IU/ml)		*p	HBeAg		*p	HBeAb		*p
	< 2E + 05	≥ 2E + 05		( +)	(−)		( +)	(−)	
**Age of mother (y)**									
< 25	29(69.0)	13(31.0)	0.002	14(33.3)	28(66.7)	0.043	22(52.4)	20(47.6)	–
25–30	148(76.7)	45(23.3)	0.004	67(34.7)	126(65.3)	0.016	112(58.0)	81(42.0)	0.599
30–35	110(88.7)	14(11.3)	–	30(24.2)	94(75.8)	0.385	85(68.5)	39(31.5)	0.058
≥ 35	52(86.7)	8(13.3)	0.649	11(18.3)	49(81.7)	–	38(63.3)	22(36.7)	0.731
**Mother Education level**									
primary	5(83.3)	1(16.7)	0.882	2(33.3)	4(66.7)	0.483	5(83.3)	1(16.7)	0.397
Junior Middle	72(75.8)	23(24.2)	0.097	32(33.7)	63(66.3)	0.066	54(56.8)	41(43.2)	–
High Middle	75(85.2)	13(14.8)	–	19(21.6)	69(78.4)	–	54(61.4)	34(38.6)	0.553
Junior college	102(80.3)	25(19.7)	0.343	40(31.5)	87(68.5)	0.104	73(57.5)	54(42.5)	0.923
Undergradute	85(82.5)	18(17.5)	0.607	29(28.2)	74(71.8)	0.231	71(68.9)	32(31.1)	0.086
**Mother occupation**									
Farmers	79(82.3)	17(17.7)	0.676	28(29.2)	68(70.8)	0.068	54(56.3)	42(43.8)	–
civil servants	29(85.3)	5(14.7)	–	6(17.6)	28(82.4)	0.401	27(79.4)	7(20.6)	0.017
worker	19(73.1)	7(26.9)	0.241	6(23.1)	20(76.9)	0.215	18(69.2)	8(30.8)	0.274
customer service staff	17(81.0)	4(19.0)	0.729	6(28.6)	15(71.4)	–	13(61.9)	8(38.1)	0.592
medical staff	10(83.3)	2(16.7)	0.871	4(33.3)	8(66.7)	0.130	7(58.3)	5(41.7)	0.952
Others	185(80.4)	45(19.6)	0.534	72(31.3)	158(68.7)	0.027	138(60.0)	92(40.0)	0.607
**Ethnic**									
Han	336(81.0)	79(19.0)	0.751	122(29.4)	293(70.6)	0.583	254(61.2)	161(38.8)	1.000
Others	3(75.0)	1(25.0)		(0)	4(100)		3(75.0)	1(25.0)	
**Fisrt known HBV infection in this test**									
Yes	71(79.8)	18(20.2)	0.751	26(29.2)	63(70.8)	0.990	53(59.6)	36(40.4)	0.683
No	268(81.2)	62(18.8)		96(29.1)	234(70.9)		204(61.8)	126(38.2)	
**Medicine during perinatal**									
Yes	94(82.5)	20(17.5)	0.643	42(36.8)	72(63.2)	0.034	61(53.5)	53(46.5)	0.044
No	245(80.3)	60(19.7)		80(26.2)	225(73.8)		196(64.3)	109(35.7)	

*The chi-square test used to compare to with the smallest percentage value.

### Blood sampling and testing of pregnant women

In order to detect HBV DNA, HBsAg, HBeAg, and HBeAb, 5 ml blood samples were collected from 419 pregnant women. The results revealed that all the pregnant women were HBsAg positive. Among them, 339 (80.9%, 339/419) had serum HBV DNA lower than 2 × 10^5^ IU/mL while the remaining 80 (19.1%, 80/419) had HBV DNA higher than 2 × 10^5^ IU/mL. HBV DNA levels tend to decrease with increasing age. The HBV DNA levels of 31% (13/42) of pregnant women aged < 25 years and 11.3% (14/124) of pregnant women aged ≥ 30 were higher than 2 × 10^5^ IU/mL. Although the HBV DNA level in pregnant women aged ≥ 35 years was higher than that of those aged 30–35 years, the difference was not statistically significant (*P* = 0.649).

Civil servants had the lowest HBV DNA levels, but there were no significant differences between them and the other occupational groups. HBV DNA levels were highest in pregnant women with high school education, but the differences between different groups were not statistically significant. There were no significant differences in the distribution of HBV DNA between different ethnic groups, regardless of whether they received antiviral treatment during pregnancy.

The overall positive rate of HBeAg was 29.1% (122/419), with the positive rate decreasing with increasing age. The positive rate of HBeAg in pregnant women of the ≥ 35-year-old group was 18.3% (11/60), while the positive rate of HBeAg in the < 25-year-old group was 33.3% (14/42), with a significant difference between both groups (*P* = 0.043). The positive ratio of HBeAg in the 25–30-year-old group was 34.7% (67/193), with a significant difference (*P* = 0.016) compared to the ≥ 35-year-old group. Pregnant women with junior high school education had the highest positive ratio of HBeAg (33.7%, 32/95), but the difference was not significant compared to the other groups. Furthermore, the highest positive rate of HBeAg was found in medical staff (33.3%, 4/12), but the difference was not significant compared to the other groups. Among the 122 HBeAg-positive women, 42 received antiviral therapy during pregnancy. The HBeAg positive rate of the group receiving antiviral treatment during pregnancy was 36.8%, which was significantly higher (*P* = 0.034) than the untreated group (26.2%).

The overall positive rate of HBeAb was 61.3% (257/419), with a gradual increase in positive rate with increasing age. The < 25-year-old group had the lowest positive rate (52.4%, 22/42), but there were no statistically significant differences between them and the other age groups. The positive rate of HBeAb was the lowest in the group with junior high school education (56.8%, 54/95), but the difference was not significant compared to the other education groups. Farmers had the lowest positive rate of HBeAb (56.3%, 54/96), while civil servants had the highest (79.4%,27/34), and the difference between them was significant (*P* = 0.017). The positive rate of HBeAb in the antiviral treated group was significantly lower (*P* = 0.044) than the non-treated group (53.5% vs. 64.3%).

### Newborn vaccination, blood sample collection, and testing

All the newborns received their first dose of 20 μg hepatitis B vaccine within 12 h of birth. The second dose was administered to 284 infants within 28–35 days, and 146 infants after 35 days. For the third dose, 369 infants were immunized within 180–210 days, 54 infants were immunized over 210 days, and 3 infants were immunized 90 days after birth, while there was no information on 2 infants. One month after the third dose was administered, blood samples were collected from a total of 319 infants. Six infants were HBsAg positive (1.9%, 6/319), while the others were HBsAg-negative (98.1%). All six HBsAg positive infants born to HBeAg postive mother (6.2%,6/97). Antiviral therapy was given to 41 HBeAg-positive pregnant women during their pregnancy, with one case of MTCT (2.4%, 1/41). Among the 80 HBeAg-positive pregnant women who did not receive antiviral therapy, five women failed to PMTCT (6.3%, 5/80) and there was no significant difference between the two groups (χ2 = 0.882, *P* = 0.348). Out of the 313 HBsAg negative infants, 300 (95.8%, 300/313) were HBsAb positive (HBsAb ≥ 10 mIU/mL) and the geometric mean concentration (GMC) of their antibody was 861.6 mIU/mL. GMC levels higher than 100 mIU/ml were detected in 260 infants (83.1%, 260/313). The highest concentration of HBsAb was 73,150 mIU/mL.

### Changes in antibody attenuation among neonates

**Figure 2:**
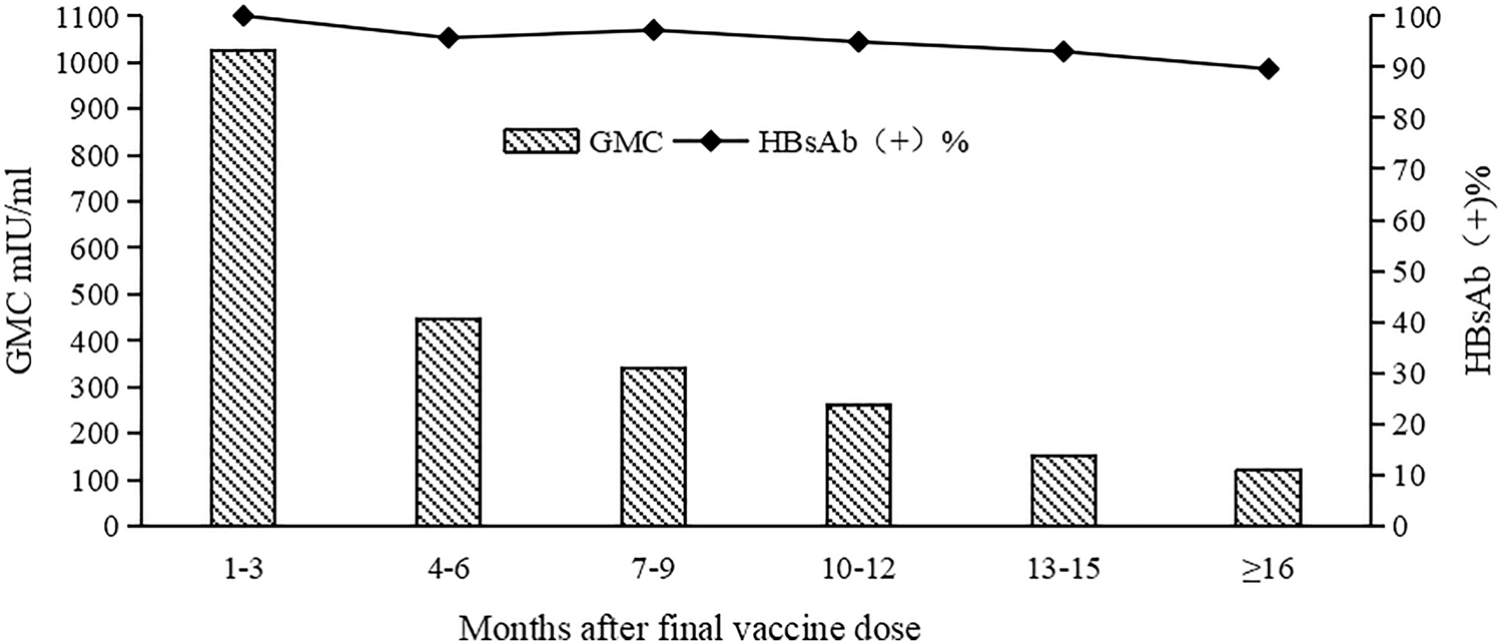
Proportion of infants with anti-HBs ≥ 10 mIU/mL with increasing interval from final vaccine dose.

A cross-sectional survey was conducted on all children who could be contacted and had received three doses of the CHO vaccine. A total of 398 blood samples were collected to detect HBsAg and HBsAb, with all children testing negative for HBsAg, while there was a decrease in HBsAb concentration among 196 children (68.3%, 196/287). The GMC of HBsAb of 398 children decreased gradually over time after completing the vaccination program. At 1–3 months after full inoculation, the GMC was 1025 mIU/ml and the HBsAb positive rate was 100%. In the population older than 16 months, the GMC decreased to 122 mIU/ml and the HBsAb positive rate decreased to 89.6% (Fig. 2). Statistical analysis revealed that there was no significant difference in HBsAb positive percent between the space-time groups (χ2 = 6.095, *P* = 0.297).

The findings showed that there was no significant link between HBsAb concentration gradient in infants and the HBV infection status or HBV DNA load in pregnant women (Table2).

**Table 2:** The distribution character of Positive-HBsAb (≥ 10mIU/ml) children with different HBV infection status of their mother.

Catagory	HBsAb (mIU/ml)	HBsAb(mIU/ml）	Χ^2^	*P*
Pregant women	10–100	≥ 100		
**HBeAg**				
Pos	14(35.0)	73(28.1)	0.807	0.369
Neg	26(65.0)	187(71.9)		
**HBV DNA(IU/ml)**				
≥ 2E + 05	9(22.5)	46(17.7)	0.535	0.462
< 2E + 05	31(77.5)	214(82.3)		
**Age(y)**				
≥ 30	17(42.5)	112(30.8)	0.005	0.945
< 30	23(57.5)	148(69.2)		

### Characteristics of HBsAg positive children

The mothers of six HBsAg positive children were all strongly positive for HBeAg, with five of them having HBV DNA loads higher than 5 × 10^8^ IU/mL. The HBV DNA load in one mother who received antiviral therapy during pregnancy was 2.13 × 10^5^ IU/ml. All six children received 20 μg CHO hepatitis B vaccine and HBIG according to schedule after birth, as well as completing all three doses of the hepatitis B vaccine (Table 3). However, two children received their second dose later, at 57 and 59 days after birth, respectively. None of the six children were underweight.

**Table 3:** Characteristics of six HBsAg-positive Children born to HBsAg-positive mother in this study.

Catagory		Value					
	Children No	1	2	3	4	5	6
Mother	HBeAg	+	+	+	+	+	+
	HBV DNA (IU/ml)	> 5.0E + 8	2.13E + 05	> 5.0E + 8	> 5.0E + 8	> 5.0E + 8	> 5.0E + 8
	Antiviral treatment	No	Yes	No	No	No	No
	age (y)	31	27	25	35	25	22
Children	Gender	F	F	F	M	M	M
	Birth weight (g)	3200	3100	3300	3700	3590	3400
	Birth dose HepB time	< 12 h	< 12 h	< 12 h	< 12 h	< 12 h	< 12 h
	HBIG time	< 12 h	< 12 h	< 12 h	< 12 h	< 12 h	< 12 h
	The second dose HepB time	31d	31d	33d	57d	31d	59d
	The third dose HepB time	184d	189d	193d	202d	209d	210d

### Reimmunization of children with low antibody levels

If the HBsAb concentration was lower than 10 mIU/ml when the sero-sample was collected the first time, three additional doses of HepB were administered. At the time of the first sero-sample, a total of 13 children were HBsAg negative and had HBsAb levels lower than 10 mIU/ml. During the second blood collection after the booster immunization, a total of ten sero-samples were collected. There were elevated HBsAb levels in 6 children (60%, 6/10), with a maximum of 681 mIU/ml and a minimum of 60.9 mIU/ml, while four children had negative HBsAb and HBsAg (40%, 4/10) (Table 4).

**Table 4:** Characteristics booster immune children born to HBsAg-positive mother in this study.

Catagory		Children									
	Childern No	1	2	3	4	5	6	7	8	9	10
Children	Primary immune (mIU/ml)	9.20	9.02	8.27	5.32	3.51	2.96	2.89	1.88	1.04	0.23
	Booster immune (mIU/ml)	1.47	4.66	1.44	3.16	181.46	60.19	216.00	90.04	681.32	323.08
	Birth weight	4200.00	3000.00	3250.00	3450.00	3350.00	3200.00	2800.00	2450.00	2700.00	3750.00
Mother	age (y)	28	26	32	29	26	33	27	30	30	29
	HBeAg	Nec	Nec	Pos	Nec	Nec	Nec	Nec	Nec	Nec	Pos
	HBeAb	Nec	Pos	Nec	Pos	Pos	Pos	Nec	Pos	Pos	Nec
	HBV DNA level (IU/ml)	< 2E + 05	≥ 2E + 05	< 2E + 05	< 2E + 05	< 2E + 05	< 2E + 05	< 2E + 05	< 2E + 05	< 2E + 05	≥ 2E + 05

## Discussion

CHB is a major public health problem that affects around 257 million people worldwide, including 65 million women of reproductive age, and causes 800,000 deaths yearly. Adults infected with HBV often have an acute infection, that leads to a self-limited disease; only 2 to 3% of them will develop CHB. However, infants with acute infection have a 95% risk of developing CHB. As a result, vertical mother-to-child transmission from HBsAg-positive mothers accounts for approximately 50% of the global disease burden^13^. There are two main ways of MTCT, one is intrauterine transmission that occurs during pregnancy and the other is blood transmission that occurs during and after delivery. WHO recommends that HBsAg-positive pregnant women with higher HBV DNA or positive HBeAg should receive antiviral treatment in order to reduce intrauterine transmission. WHO also recommends that all infants should receive a birth dose of the hepatitis B vaccine immediately after birth, followed by two further doses to complete the primary series of vaccinations.

Since the 1990s, HBV vaccination in children has been widely implemented in more than 200 countries, leading to significant reductions in MTCT and global CHB incidence. Previous reports have shown that HBV transmission in newborns of HBeAg-negative mothers can be almost completely inhibited by the timely completion of the whole HBV vaccination course^14^, however, about 10% newborns born to HBsAg and HBeAg double positive mothers may be infected with HBV through MTCT. In previous studies, HBsAg antigen expressed by CHO cells was found to be more stable^15^, and the immune effect was better than that of yeast after use in adults^16^. Furthermore, vaccines prepared with HBsAg containing PreS1 expressed in CHO have a good immune response in immunodeficient persons^17^. Therefore, it may also be interesting to understand the effects of HepB with high concentration derived from CHO cells on MTCT prevention. The results of this study showed that administration of three doses of 20 μg HepB (plus HBIG) achieved a totally prevention success rate of 98.1% and the HBeAg-negtive mother achieved 100% prevention and HBeAg-positive mother achieved 93.8% for prevention success rate. Previous retrospective study had showed that 5 μg HepB (plus HBIG) achieved 94.4% and the 10 μg achieved 96.5% on blocking MTCT^18,19^. 20 μg HepB (plus HBIG) derived from CHO cell seems to have a greater advantage on preventing MTCT. WHO had set the goal of eliminating hepatitis on the whole world by 2030 in 2021, including MTCT blocking rate reached to 98% by the way of vaccination^20^. So finding more effective vaccine was also a daunting task.

The occurrence of HBV MTCT is closely related to the presence of HBV infection in the mother. Several studies reported that pregnant women who were HBeAg positive or had an HBV DNA level higher than 2 × 10^5^ IU/ml had a significantly increased probability of MTCT^4,21^. According to the 2014 HBV sero-survey of China, the average HBsAg positive rate among Chinese women under the age of 29 was 2.6%^22^. Due to the differences in economy and lifestyle, women of childbearing age living in different provinces of China often have varying HBsAg-positive rates. In certain provinces in southeastern China, the HBsAg positive rate among pregnant women aged 15–49 years is as high as 11.2%^12^. In 2012, a 3.7% HBsAg positive rate among women of childbearing age in the central Chinese province of Henan was reported^23^.

In this study, the positive rate of HBeAg in HBsAg-positive pregnant women was 29.1% in China, whereas the positive rate ranged from 11.2 to 35.1% in other previously published studies^24–26^. In some European countries, the rate was considerably higher than 50%^27,28^. Many studies have reported that the positive rate of HBeAg in HBsAg-positive women significantly decreases with increasing age. Our findings were consistent with previous studies, as we observed a gradual decrease in the positive rate of HBeAg with the increasing age of pregnant women. In this study, the success rate of PMTCT of infants born to HBeAg-positive pregnant women was 93.8%, and no children born to HBeAg-negative pregnant women were infected. Six mothers who gave birth to children with positive HBsAg and negative HBsAb had HBV DNA levels higher than 2 × 10^5^ IU/ml, with five of them having levels higher than 5 × 10^8^ IU/ml. Maternal HBeAg positivity and an increased HBV DNA level were risk factors for HBV MTCT. As previously mentioned, maternal HBeAg positivity may even be an independent risk factor^29^. Previous study had found that more than 1% of HBV infections in newborns who had vaccinated timely were due to intrauterine infections. However, positive HBeAg and higher HBV DNA level of pregnant women were risk factors related to intrauterine infections. Therefore, more attention should also be paid to health education of pregnant women. Timely and continuously antiviral treatment before pregnancy, in order to reduce HBV DNA level, and even realize the serological conversion of HBeAg, may be more meaningful for improving the blocking rate of mother-to-child infection.

Previous studies have reported that antiviral therapy during pregnancy can effectively reduce the serum HBV DNA level, even reaching the serological conversion of HBeAg and HBeAb^30,31^. In this study, the HBeAg positive rate of pregnant women who received antiviral therapy was significantly higher (*P* = 0.034) than those who did not (36.8% VS 26.4%). This was potentially due to people who received antiviral treatment having more severe clinical symptoms, i.e., they had positive HBeAg and a higher HBV DNA level. The antiviral treatment aimed to treat CHB rather than prevent MTCT. Among the six cases that failed to prevent MTCT, one pregnant woman received antiviral therapy during her pregnancy (16.7%, 1/6), but her HBV DNA level was still higher than 2 × 10^5^ IU/ml. There were no significant differences in MTCT between the group receiving antiviral treatment and those who did not. The treatment procedures for pregnant women to evaluate the effect of MTCT, rather than the care procedure for CHB in general, still require improvement. On the other hand, the sample size of the preventing failure of MTCT was very small in this study, which couldn’t truly reflect the therapeutic effect and may bring large sampling error. So perhaps a larger sample size is necessary to get real results.

The HBsAb titer of children was an important indicator to determine whether they possessed immunity to HBV infection. In this study, the GMC of HBsAb in children with successful HBV prevention was not associated with the maternal HBV infection status. One month after the complete immunization, the GMC was 861.6mIU/mL. The GMC of HBsAb gradually decreased with increasing age, and 17 months after the entire immunization process, the positive rate of HBsAb also decreased from 100 to 89.1%. Federica Chiara et al. had showed that the HepB administered before one year of age tends to cause a significant decrease in the positive rate and the GMT of HBsAb^32^. It is probably related to the differences between innate immunity and adaptive immunity. Other finding showed that if the response to the HepB is positive, the decrease of the positive rate of HBsAb even below 10 mIU/ml does not mean a loss of protection^33^. But for the infants with HBsAg-postitive mother, a post-vaccination serological test (PVST) was recommended before the occurrence of a rapid decline 4–6 months following complete immunization. According to WHO guidelines, infants whose mothers are HBsAg positive can receive three injections of enhanced immunization if they are negative for both HBsAg and HBsAb after the immunization program. In this study, ten children who had HBsAb lower than 10 mIU/ml after completing the vaccination program received another three doses of 20 μg HepB as a booster. Although six children had HBsAb higher than 10 mIU/ml, the other four still had HBsAb lower than 10 mIU/ml. These results suggested that timely detection of antibodies after complete immunization, as well as revaccination for children with HBsAb lower than 10 mIU/ml, were critical for the success of PMTCT.

## Conclusion

20 μg HepB was effective in preventing vertical transmission of HBV. It is crucial to enhance health education for childbearing age women so that they may participate in early HBsAg screening and be aware of their HBV infection status. Although several technical guidelines have recommended antiviral therapy for HBsAg-HBeAg positive pregnant women or those with a higher HBV DNA level, a more viable treatment procedure for greater prevention of HBV MTCT is still lacking in China, even worldwide.

## Material and methods

### Study population

The Xinzheng county and Jiefang district of Jiaozuo in Henan province in central China were selected as the survey points based on the previous prevalence rate of HBsAg-positive women of childbearing age. All HBsAg-positive pregnant women who gave birth between May 1, 2018, and Oct 31, 2019, in the two areas were interviewed. Their infants (those that survived) were vaccinated with three doses of 20 μg HepB at intervals of 0, 1, and 6 months, as well as one dose of 100 IU HBIG, all of which were provided for free.

### The HBV infection status and information of HBsAg-positive women

Pregnant women were screened for HBsAg using the ELISA method when they visited the hospital for perinatal health care in China. All the pregnant women who participated in the survey were interviewed and given a questionnaire to fill out. Personal information about the pregnant women, such as age, gestational age, history of therapy, and other factors, were included in the questionnaire. Before delivery, 5 ml of blood sample was taken from the expecting mothers. All serum samples were delivered to the Henan provincial Center for Disease Control and Prevention to test for HBsAg, HBeAg, and HBeAb using ELISA test kits (Beijing Wantai Biological Pharmacy Enterprise Co., Ltd.). The amount of HBV DNA in the collected serum was measured by real-time PCR using the HBV DNA test kit (DAAN Gene Co., Ltd. China).

### The vaccination and serology test of infants

The HepB used in this study contained 20 μg HBsAg in each dose (Jintan Biotechnology Co., Ltd, China). All newborns received the birth dose vaccine and 100 IU HBIG within 12 h after birth, followed by the second dose vaccine within 28–35 days after birth, and the third dose vaccine between 180 and 210 days after birth. One month after completing the basic immunization program, 3 ml blood samples (the first blood samples) were collected to test for HBsAb and HBsAg. In June 2020, 3 ml blood samples (the second blood samples) were collected from all enrolled HBsAg negative infants to detect HBsAb and HBsAg.

### Statistical analysis

All data were double entered into an EPI Data 3.02 software database and cross-checked against the original records. After accuracy verification, data were analyzed at HNCDC using the SPSS 19.0 software for Windows (SPSS Inc., Chicago. IL). The sociodemographic characteristics of the participants and their infection status in terms of HBsAg positivity were described using proportions and ratios. The chi-square test or Fisher exact probability test was used to determine the statistical significance of the associations, with a significance level of *P* < 0.05. The chi-square analysis was used to analyze the differences.

### Ethical approval statement

The survey was approved by the Henan provincial Center for Disease Control and Prevention Ethics Committee. Each participant was informed of the survey’s purpose, as well as their right to have their information kept confidential. The informed consent was obtained as following: the pregnant women's informed consent were signed and confirmed by themself and the informed consent of the newborns were signed by their parents (or other legal guardians entrusted by their parents). In addition, all interviews, laboratory tests, and notification of results were provided free of charge to the participants. The test results were enveloped, sealed, and personally delivered to each participant by the local doctor. This study was performed in accordance with relevant guidelines and regulations.

## Data Availability

All data generated or analysed during this study are included in this published article.
